# Supplementary material for: Development, Content Validity and Usability of a Self-Assessment Instrument for the Lifestyle of Breast Cancer Survivors in Brazil
Source: Nutrients. 2024 Oct 30;16(21):3707. doi: 10.3390/nu16213707 (PMC11547887; doi:10.3390/nu16213707)
Supplement: Supplementary file 1 [file nutrients-16-03707-s001.zip › Table S1.pdf]

Table S1. Structure and scoring format (by component and total) of PrevCancer, a questionnaire to assess lifestyle-related risk factors for cancer prevention.

**Components and topics**	**Answer options and scoring format**	**Comments**
1. Date of birth<br>Automatic system component – age calculation (years)	(day/month/year)	Discursive answer. Automatic age calculation based on the question (current date – date of birth = current age). The age component will be important at the beginning of the instrument to enable the correct classification of BMI afterwards.
Component 1: Weight and height		
2. Current body weight (Kg)	_______kg	Current body weight: Discursive answer. It will allow the automatic calculation of the BMI. The system allows locks (minimum and maximum body weight). Minimum: 10 kg. Maximum: 300 kg. Only values in numbers will be allowed. Question justification: "*Weight and height component*" component, involving body weight, height and BMI, based on recommendations already established in the literature [7,18]
3. Height (m).	____________m	Height: Discursive answer. It will allow the automatic calculation of the BMI.<br>The system allows locks (minimum and maximum height). Minimum: 1 m. Maximum: 2.5 m. Only values in numbers will be allowed. Question justification: "*Weight and height component*" component, involving body weight, height and BMI, based on recommendations already established in the literature [7,18]. The system will allow two places after the decimal point.
Automatic BMI calculation (Kg/m^2)	*BMI Automatic Score:*<br>*Patients up to 59 years of age (adults):*<br>*- Between 18.5 and 24.9 Kg/m2 (0.5 point)*	

	- Between 25 and 29.99 Kg/m2 (0.25 point)<br>*- <18.5 or ≥30 Kg/m2 (0 point)*<br><br>*Patients 60 years of age or older (elderly):*<br>*- Between 23 and < 28 Kg/m2 (0.5 point)*<br>*- ≥ 28 and < 30 Kg/m2 (0.25 point)*<br>*- <23 or ≥ 30 Kg/m2 (0 point)*	Automatic calculation (system): Weight / Height / Height = BMI (Kg/m2). Score rationale: "*Weight and height component*" component, involving body weight, height, and BMI, based on recommendations already established in the literature [7,18]
Component 2: Body image<br><br>4. Body image	( ) 1<br>( ) 2<br>( ) 3<br>( ) 4<br>( ) 5<br>( ) 6<br>( ) 7<br>( ) 8<br>( ) 9<br><br>*Stunkard Silhouette Scale Score:*<br>*- Figures numbered 1 to 5, normal weight (0.5 point)*<br>*- Figures 6 and 7, overweight (0.25 point)*<br>*- Figures 8 and 9, obesity (0 points)*	Body image: multiple choice answer. It is suggested to consider figure 1 as "normal weight", thus gaining 0.5 points, considering that this component is more focused on excess abdominal fat, and not on thinness itself [7,18]
Component 3: Physical activity		

5. Moderate physical activity	___________ minutes per week	Moderate and vigorous/intense physical activities: Discursive answers. Rationale for the questions: "physical activity" component is based on recommendations already established in the literature [7,18]. The question was developed based on the Cancer Health Check [22]. The system allows locks (minimum and maximum practice of physical activity). Minimum: 0 minutes. Maximum: 10080 minutes. Only values in numbers will be allowed. New physical exercises were included from the American Institute for Cancer Research [48].
6. Vigorous/intense physical activity	___________ minutes per week	
<u>Automatic calculation of total duration of physical exercise practice per week (minutes)</u>	*Score of physical exercise per week (minutes)*<br>*- Moderate physical activity ≥ 150 minutes (1 point)*<br>*- Vigorous physical activity ≥ 75 minutes (1 point)*<br>*- Moderate and/or (+) vigorous physical activity = ≥ 150 minutes (1 point)*<br>*- Moderate and/or (+) vigorous physical activity = 75 to <150 minutes per week (0.5 point)*<br>*- Moderate physical activity = <75 minutes per week (0 point)*<br>*- Vigorous physical activity = 38 to <75 minutes per week (0.5 point)*	Automatic calculation: sum of the weekly minutes of physical exercise of moderate and vigorous/intense intensity (sum of answers to questions 6 and 7). Score justification: "physical activity" component is based on recommendations already established in the literature [7,18]. The 75-minute and 150-minute cutoff points were established based on a previously published assessment score for adherence to the WCRF/AICR recommendations [21].

	- Vigorous physical activity = <38 minutes per week (0 point)	
Component 4: Fruits and non-starchy vegetables		
7. Fruits consumption	_________ servings per day	Daily servings of fruits and vegetables: discursive answers.
8. *Non-starchy v*egetables consumption	_________ servings per day	In the WCRF/AICR report from a Brazilian perspective[2], legumes are not included in the 400 g daily intake of fruits and vegetables: "Consume a diet rich in all types of plant foods, including at least five servings (at least 400 grams in total) of a variety of fruits, non-starchy vegetables and greens every day).<br>In the international versions of Screener, legumes are being counted.<br>For this question, we chose to consider only non-starchy vegetables [18,21], with exemplified portions [22,52,53].<br>The examples of non-starchy vegetables selected are based on the most consumed options highlighted in the Family Budget Survey (POF) 2017-2018 report [36].
<u>Automatic calculation of total daily consumed weight of fruits, non-starchy vegetables and greens</u>	*Score of total daily consumed weight of fruits, non-starchy vegetables and leafy greens:*<br>*- ≥ 400 g of fruits, non-starchy vegetables and greens consumed daily (0.5 point)*	Calculation of total daily consumed weight of fruits, non-starchy vegetables, and greens: number of servings consumed x 80 g.<br>Score based on previously published WCRF/AICR recommendations assessment score [21].

	- Between 200 and <400 g/day of fruits, non-starchy vegetables and greens consumed (0.25 point)<br>*- <200 g/day of fruits, non-starchy vegetables and greens consumed (0 point)*	
Component 5: Beans		
9. Beans consumption	( ) Once a week or less (0 points)<br>( ) 2 to 4 times a week (0.25 points)<br>( ) 5 times a week or more (0.5 point)	Multiple choice answer. Justification of the question: question included considering the importance of beans in the Brazilian menu [49,50], and considering that beans are not included in question 8. To define the score for this question, the Food Guide for the Brazilian Population of 2008 [49], which recommends that 1 serving of legumes should be consumed daily.
Component 6: Sugary drinks		
10. Sugary drinks consumption	( ) Once a week or less (0.5 point)<br>( ) 2 to 3 times a week (0.25 points)<br>( ) 4 times or more per week (0 points)	Multiple choice answer. Justification of the question: specific recommendations regarding sugary drinks have already been published [7,18]. The alternatives are based on the international version of the Screener (WCRF Cancer Health Check) [22].
Component 7: Ultra-processed foods		
11. Ultra-processed foods consumption	( ) Once or less per week (0.5 point)<br>( ) 2 to 3 times a week (0.25 points)<br>( ) 4 times or more per week (0 points)	Multiple choice answer. Justification of the question: specific recommendations regarding ultra-processed foods have already been published [7,18,49,50,51]. The alternatives are based on the international version of the Screener (WCRF Cancer Health Check) [22].

Component 8: Whole grains, nuts and seeds		
12. Whole grains, nuts and seeds consumption	( ) I don't consume it every day (0 points)<br>( ) Once a day (0.25 point)<br>( ) 2 times or more per day (0.5 point)	Multiple choice answer. The alternatives are based on the international version of the Screener (WCRF Cancer Health Check) [22]. Justification of the question: the WCRF/AICR encourages adequate fiber intake through different food sources, which include whole grains [7,18].
Component 9: Red meat and processed meat		
13. Red meat consumption	( ) 3 servings or less per week (0.5 point)<br>( ) 4 to 5 servings per week (0.25 points)<br>( ) 6 servings or more per week (0 points)	Multiple choice answer. The alternatives are based on the international version of the Screener (WCRF Cancer Health Check) [22]. For the weight to be considered for each portion of red meat, the WCRF/AICR [7,18] recommendation of 350 to 500 g of already prepared meat was followed, which is equivalent to 3 portions of red meat (350 to 500 g / 3 = portion between 116.6 to 166.6 g).
14. Processed meat consumption	( ) Rarely or never (0.5 point)<br>( ) Once a week (0.25 point)<br>( ) 2 times a week or more (0 points)	Multiple choice answer. The alternatives are based on the international version of the Screener (WCRF Cancer Health Check) [22]. Score based on previously published score for assessing adherence to WCRF/AICR recommendations [21]. Justification for the question: WCRF/AICR recommends not consuming processed meat [7,18].
Component 10: Alcoholic drinks		
15. Alcoholic drinks consumption	( ) I do not consume alcoholic beverages	

	(.) Wine and/or sparkling wine:<br>- Specify the quantity (in numerals only):________ small glass(s) of 150 ml per week<br><br>( ) Beer<br>- Specify the quantity (in numerals only):________ 350 ml can(s) per week<br><br>( ) Draft beer<br>- Specify the quantity (in numerals only):________ 290 ml "tulip" glass(s) per week<br><br>( ) Distilled drinks (whisky, vodka, rum, cachaça, among others)<br>- Specify the quantity (in numerals only):________ dose(s) of 50 ml per week	Discursive answer. Portions of drinks according to previously consulted bibliography [52,53]. Justification of the question: the WCRF/AICR provides specific recommendations on alcoholic beverages [7,18].
<u>Automatic calculation of total daily consumption of ethanol</u>	*Score of the total daily grammage consumed of ethanol.*	For scoring, the system will consider answer to question 1 (male/female). Score based on previously published WCRF/AICR recommendations assessment score [21]. The information on

	- 0 g of ethanol (1 point)<br>*- > 0 and < 14 g/day (0.5 point)*<br>*- ≥ 14 g/day (0 point)*	the volume of daily alcohol intake (ml) will be converted into g of ethanol considering the alcohol levels established by the National Institute of Alcohol Abuse and Alcoholism [54]. The density of the ethanol that will be used is 0.8 g/cm³ [54,55].<br><br>Automatic calculation:<br>1) Number of doses x ml of portion<br>2) Total result above / 7 (7 days a week)<br>3) g ETHANOL = [(beverage volume in ml X alcohol content)/100]X ethanol density (0.8)<br>[(42.8 x 12)/100)] x 0.8 = 4.1 g of ethanol [54,55].
Component 11: Breastfeeding		
16. Sum of the months of exclusive breastfeeding	( ) I have never breastfed or have not breastfed exclusively (0 points)<br>( ) I breastfed for a maximum of 5 months in total (0.5 point)<br>( ) I breastfed for 6 months or more in total (1 point)	Multiple choice answer. <u>Rationale for the question</u>: the WCRF/AICR has a specific recommendation on breastfeeding [7,18]. Score based on previously published WCRF/AICR recommendations assessment score [21].
Component 12: Chimarrão (matte drink)		
17. "Chimarrão" (matte) consumption and temperature	( ) Yes, very hot (boiling, with bubble formation) (0 point)<br>( ) Yes, hot (0.25 point)<br>( ) Yes, warm (0.5 point)	Multiple choice answer. <u>Justification of the question</u>: from the Brazilian perspective [18], the consumption of "chimarrão"/ mate (*Ilex paraguariensis*) is common in the southern region of Brazil. Much is discussed about the consumption of very hot mate and the increased risk of some types of cancer, such as the esophageal cancer. In the context of patients with breast cancer, this

	( ) Rarely or never (0.5 point)	component was inserted to assess the risk of developing a new type of cancer. The reference of very hot mate is the formation of bubbles [18].
Component 13: Smoking habits		
18. Smoking habits	( ) Yes, I currently smoke (0 points)<br>( ) No, but I am a former smoker (0.5 point)<br>( ) No, I never smoked (1 point)	Multiple choice answer. The alternatives are based on the international version of the Screener (WCRF Cancer Health Check) [22] and Global Tobacco Surveillance System [56]. Question rationale: The WCRF/AICR provides additional recommendations on avoiding smoking [7,18].
Component 14: Sun exposure		
19. Daily sun exposure	( ) Yes, and my arms, legs and/or back are directly exposed to the sun and I do not use sunscreen (0 points)<br>( ) Yes, and I cover my arms, legs and/or back so as not to be directly exposed to the sun and/or I use sunscreen (0.5 point)<br>( ) No (1 point)	Multiple choice answer. The alternatives are based on the Sun questionnaire Exposure and Protection Index, validated and translated into Portuguese [57]. Rationale for the question: the WCRF/AICR brings additional recommendation on avoiding inadequate sun exposure [7,18].
**20. Full name**	-	Discursive answer.
**21. Email**	-	Discursive answer.
**22. Telehone**	Region code + phone	Discursive answer.
**23. City of origin**	-	Multiple choice answer. In the system, a list of city options will be included according to the selected state.
**24. Skin color (ethnicity)**	( ) White<br>( ) Black<br>( ) Brown	Multiple choice answer. Classified according to Brazilian Institute of Geography and Statistics [58]

	( ) Indigenous<br>( ) Yellow	
**25. Education**	( ) I did not study at school<br>( ) 1st grade (elementary school) incomplete<br>( ) 1st degree (elementary school) completed<br>( ) Incomplete 2nd degree (high school)<br>( ) 2nd degree (high school) complete<br>( ) Higher education (undergraduate) incomplete<br>( ) Higher education (undergraduate) completed<br>( ) Graduate (graduation, post post-graduation) completed	Multiple choice answer.
**26. Occupation**	-	Multiple choice answer. In the system, a list of profession options will be included.
**27. Family's average monthly income**	( ) Up to 2 minimum wages<br>( ) More than 2 to 5 minimum wages<br>( ) More than 5 to 10 minimum wages<br>( ) More than 10 to 20 minimum wages<br>( ) More than 20 minimum wages	Multiple choice answer. Classified according to Brazilian Institute of Geography and Statistics [58,59]
**28. Marital status**	( ) Married<br>( ) Stable union<br>( ) Widow<br>( ) Separated<br>( ) Single	Multiple choice answer.
**RESULT:**<br>Total score = 0 to 10 points<br><br>0 to 3 points – unhealthy lifestyle		

Attention! Your lifestyle habits are unhealthy, which can increase your risk of developing new cancer or having cancer return.

4 to 7 points – moderately healthy lifestyle

Cool! You already follow some healthy habits that help prevent the development of new cancer or the recurrence of cancer. However, your lifestyle can be even better by following healthy lifestyle guidelines.

8 to 10 points – healthy lifestyle

Congratulations! You follow a healthy lifestyle that helps prevent the development of new cancer or the recurrence of cancer. Continue to have a healthy body weight, eat healthy, practice physical activity and avoid smoking and inadequate sun exposure.

7. World Cancer Research Fund/American Institute for Cancer Research. Diet, Nutrition, Physical Activity and Cancer: A Global Perspective. Continuous Update Project Expert Report 2018. Available online: https://www.wcrf.org/wp-content/uploads/2021/02/Summary-of-Third-Expert-Report-2018.pdf (accessed on 1 September 2019). 18. Brazil. Ministry of Health. José Alencar Gomes da Silva National Cancer Institute. *Dieta, Nutrição, Atividade Física e Câncer: Uma Perspectiva Global: Um Resumo do Terceiro Relatório de Especialistas Com uma Perspectiva Brasileira*; José Alencar Gomes da Silva National Cancer Institute: Rio de Janeiro, Brazil, 2020. 21. Shams-White, M.M.; Brockton, N.T.; Mitrou, P.; Romaguera, D.; Brown, S.; Bender, A.; Kahle, L.L.; Reedy, J. Operationalizing the 2018 World Cancer Research Fund/American Institute for Cancer Research (WCRF/AICR) cancer prevention recommendations: A standardized scoring system. *Nutrients* **2019**, *11*, 1572. https://doi.org/10.3390/nu11071572. 22. American Institute for Cancer Research. Cancer Health Check. Available online: https://www.aicr.org/cancer-health-check/ (accessed on 16 August 2024). 36. Brazilian Institute of Geography and Statistics. *Family Budget Survey 2017–2018: Analysis of Personal Food Consumption in Brazil*; Brazilian Institute of Geography and Statistics: Rio de Janeiro, Brazil, 2020. 48. American Institute for Cancer Research. *Living Well with Cancer and Beyond: Your Cancer Resource*; American Institute for Cancer Research: Arlington, TX, USA, 2024. 49. Brazil. Ministry of Health. Department of Health Care. Department of Primary Care. *Food Guide for the Brazilian Population*: *Promoting Healthy Eating*; Ministry of Health, Department of Health Care: Brasília, Brazil, 2008. 50. Brazil. Ministry of Health. Department of Health Care. Department of Primary Care. *Food Guide for the Brazilian Population*, 2nd ed.; Ministry of Health, Department of Health Care: Brasília, Brazil, 2014. 51. Monteiro, C.A.; Cannon, G.; Levy, R.; Moubarac, J.C.; Jaime, P.; Martins, A.P.; Canella, D.; Louzada, M.; Parra, D. NOVA. The star shines bright. *World Nutr.* **2016**, *7*, 28–38. 52. Philippi, S.T. *Tabela de Composição de Alimentos: Suporte Para Decisão Nutricional*; Manole: São Paulo, Brazil, 2013; pp. 164–164. 53. Pinheiro, A.B. *Tabela Para Avaliação de Consumo Alimentar em Medidas Caseiras*, 5th ed.; Atheneu: São Paulo, Brazil, 2008; 131p. 54. National Institute on Alcohol Abuse and Alcoholism. *Rethinking Drinking: Alcohol and Your Health*; National Institute on Alcohol Abuse and Alcoholism: Washington, DC, USA, 2024. 55. National Center for Biotechnology Information. PubChem Compound Summary for CID 702, Ethanol. Available online: https://pubchem.ncbi.nlm.nih.gov/compound/Ethanol. (accessed on 15 August 2024). 56. Global Tobacco Surveillance System; Collaborative Group for the Global Adult Smoking Survey. *Tobacco Questions for Surveys*: *A Subset of Key Questions from the Worldwide Adult Smoking Survey*, 2nd ed.; United States Centers for Disease Control and Prevention: Atlanta, GA, USA, 2011. 57. Detert, H.; Hedlund, S.; Anderson, C.; Rodvall, Y.; Festin, K.; Whiteman, D.; Falk, M. Validation of sun exposure and protection index (SEPI) for estimation of sun habits. *Cancer Epidemiol*. **2015**, *39*, 986–993. https://doi.org/10.1016/j.canep.2015.10.022. 58. Brazil. Ministry of Health. *Race/Color/Ethnicity Implementation Guide;* Ministry of Health: Brasília, Brazil, 2018. 59. Brazilian Institute of Geography and Statistics. *Synthesis of Social Indicators: An Analysis of the Living Conditions of the Brazilian Population*; Brazilian Institute of Geography and Statistics—Population Coordination and Social Indicators: Rio de Janeiro, Brazil, 2023.
