# Supplementary material for: Development, Content Validity and Usability of a Self-Assessment Instrument for the Lifestyle of Breast Cancer Survivors in Brazil
Source: Nutrients. 2024 Oct 30;16(21):3707. doi: 10.3390/nu16213707 (PMC11547887; doi:10.3390/nu16213707)
Supplement: Supplementary file 1 [file nutrients-16-03707-s001.zip › Table S2.pdf]

Table S2. Example of automatic feedback after completing PrevCancer instrument.

SPECIFIC GUIDELINES

For each component of the questionnaire, it will be presented, in the form of a report, how much the participant follows or does not follow the recommendation. Illustrative images will demonstrate how each recommendation is being followed, as shown below.

"To check how well you are following healthy lifestyle recommendations, consider the following classification below:"

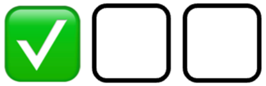 Low adherence to the recommendation

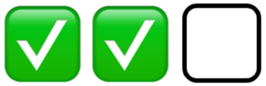 Moderate adherence to the recommendation

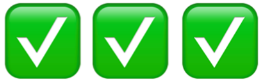 High adherence to the recommendation

**Degree of follow-up of the recommendation**		**Recommendations**
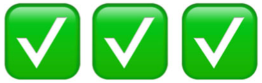	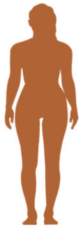	**Body weight:** people who are obese or below adequate body weight are more likely to develop different types of diseases, including cancer. It is important that you have follow-up with a health professional so that you can monitor your body weight and, together, define strategies for you to follow so that your weight can remain adequate.
		**Physical activity:** Being more active will bring many benefits to your health and will improve your prevention against diseases such as cancer. It is recommended that you perform 150 minutes per week of moderate-intensity physical exercise, such as walking, cycling, weight training, housework,

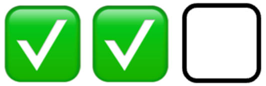 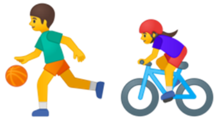

gardening, swimming, dancing, Pilates, Zumba, yoga, or any activity that moderately increases your breathing or heart rate. from heart. These 150 minutes of physical activity per week can be achieved, for example, by performing 30 minutes of physical exercise 5 times a week, or 50 minutes of physical exercise 3 times a week. To achieve the physical activity recommendation, you can also choose to perform 75 minutes per week of more intense physical exercise, such as running, brisk swimming, brisk cycling, aerobics, some group sports, or any activity that greatly increases your blood pressure. breathing or heartbeat. These 75 minutes per week can be achieved, for example, by performing 25 minutes of intense physical exercise 3 times a week.

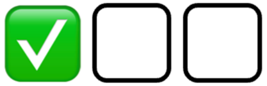 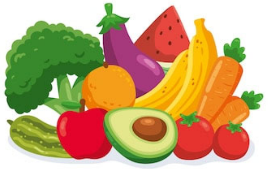

Consumption of fruits, vegetables and greens: To guarantee the nutrients necessary for your health, it is recommended that you eat a diet rich in foods such as fruits, vegetables and greens every day. Ideally, you should consume around 5 portions of these foods daily, with each portion being approximately the size of the palm of your closed hand (80 grams). Examples of a serving of fruit include 1 medium banana, or 1 small orange/tangerine, or 1 small slice of watermelon, or 1 small apple. Examples of vegetables are 2 serving spoons of sliced carrots, or 2 serving spoons of cooked cabbage, or 2 serving spoons of cooked chayote, or 5 tomato slices, or 1 full plate/bowl of leaves such as lettuce and arugula. Vegetables do not include, in this recommendation, starchy foods such as potatoes, sweet potatoes, cassava and yams. This recommendation also does not include dried fruits and legumes such as beans, lentils and chickpeas.

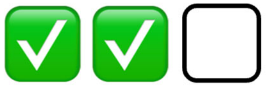 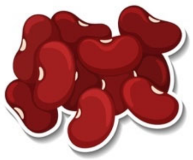

Consumption of beans: Beans are a group of foods that include, for example, all colors of beans, lentils and chickpeas. These foods are rich in proteins, dietary fiber, vitamins, iron, calcium and other nutrients important for your health. It is recommended that you consume 1 serving of legumes daily, such as 1 scoop of beans, or 2 serving spoons of chickpeas, or 1 scoop of lentils.

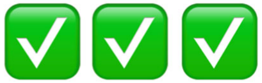 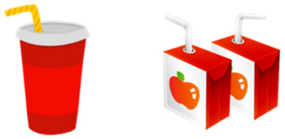

Sugary drinks: Sugary drinks should be avoided, as they are not very nutritious and increase the chance of gaining weight inappropriately. Examples of sugary drinks are soft drinks, ready-to-drink processed juices with sugar (“box juices” or “powdered juices”), natural juices with added sugar, ready-to-drink industrialized teas (such as industrialized mate tea in a bottle or can) , coffee

and tea with added sugar, and energy/sports drinks. Try swapping sugary drinks for water and fruit.

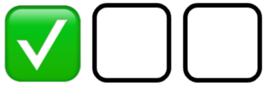

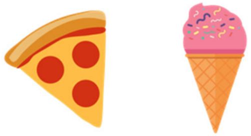

Industrialized (ultra-processed) foods: Choose more natural foods, such as fruits, vegetables, whole grains and legumes, and avoid processed foods such as ready-to-eat snacks, fried foods, desserts and sweets. Some examples of processed foods that should be avoided are stuffed biscuits, packaged snacks, instant noodles, ice cream, chocolates, breakfast cereals, fast food foods such as pizzas and French fries.

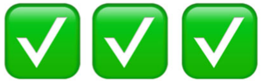

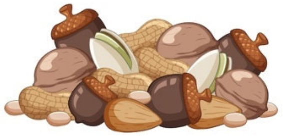

Whole grains, nuts and seeds: Foods from the whole grain group, as well as nuts and seeds, contain several important nutrients for your health and cancer prevention. Examples of cereals, nuts and seeds are: whole grain bread, wholemeal flour, brown rice, wholemeal pasta, oats, flaxseed, chia, quinoa, sunflower seeds, pumpkin seeds, Brazil nuts, cashew nuts, walnuts and almonds. Try to include these types of foods twice or more a day.

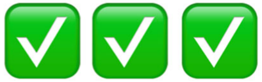

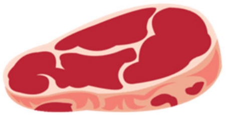

Red meat: Although meat is a source of protein, iron and some vitamins, such as complex B, the naturally present fat and chemicals generated during processing are harmful to health. Therefore, consume red meat a maximum of 3 times a week to avoid harm to your health due to this consumption. Examples of a serving of red meat include: 1 large beef steak, or 2 serving spoons of ground beef, or 1 medium slice of lamb, or 1 roast pork chop, or 2 slices of roast loin.

✅✅☐	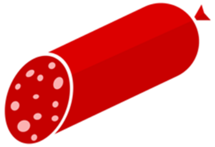	**Processed meat:** Processed meat, such as industrialized "fast food" hamburgers, bacon, sausages, sausages, salami, ham, smoked turkey breast, mortadella, and other embedded foods, contains chemicals generated during processing that are harmful to health and increase the risk of cancer. Avoid consuming these types of foods as much as possible.
✅✅✅	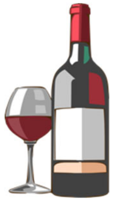	**Alcoholic drinks:** It is recommended that alcohol consumption be avoided, especially if the intention is to prevent cancer. Even in small amounts, alcohol can increase your risk of cancer. Some examples of alcoholic drinks include cachaça, draft beer, beer, vodka, wine, sparkling wine, whiskey, and rum.
✅☐☐	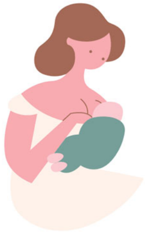	**Breastfeeding:** Breastfeeding is an important way of protecting the mother against breast and ovarian cancer, for example. The longer the mother can breastfeed, the greater the protection for the mother. The baby also benefits from breastfeeding, as it is the child's first healthy eating strategy, guaranteeing the nutrients necessary for the beginning of life. It is recommended that breastfeeding be carried out exclusively until the child is six months old, and until the child is two years old in a complementary manner.
✅✅☐	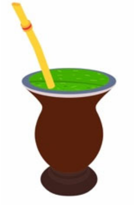	**Consumption of "chimarrão"/ mate:** Consumption of "chimarrão"/mate at very high temperatures is related to the development of some types of cancer, mainly esophageal cancer. Therefore, it is recommended, to prevent cancer, that the temperature of the drink is below 60 °C. To reach this temperature, it is advisable to turn off the heat when gaseous bubbles start to form at the bottom of the pan or kettle and wait a few minutes before consuming the drink.

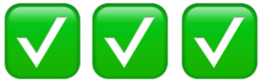

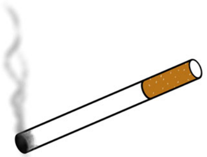

Smoking: The chemical substances released by cigarettes in their different forms (conventional cigarettes, straw, cigars, among others) increase the risk of various types of cancer. Therefore, smoking should be avoided, as well as excessive exposure to smoke.

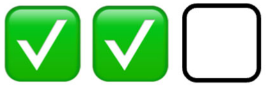

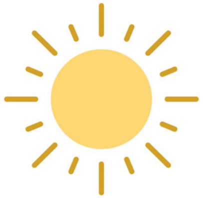

Sun exposure: Inadequate sun exposure, that is, at peak sunlight hours (between 10 am and 4 pm) and without adequate skin protection (without using sunscreen and arms, legs and/or back exposed to the sun) can increase inflammation and the risk of some types of cancer. Therefore, expose yourself to the sun for about 10 to 15 minutes a day at times that are not peak sunlight. If you spend more than 10 to 15 minutes in the sun and/or are exposed during peak sunlight hours, apply sunscreen with a protection factor (SPF) of 15 or more.

Your final score:

8.5 / 10

Healthy lifestyle

Congratulations! You follow a healthy lifestyle that helps prevent the development of new cancer or the recurrence of cancer. Continue to have a healthy body weight, eat healthy, practice physical activity and avoid smoking and inadequate sun exposure.
