# Supplementary material for: Development, Content Validity and Usability of a Self-Assessment Instrument for the Lifestyle of Breast Cancer Survivors in Brazil
Source: Nutrients. 2024 Oct 30;16(21):3707. doi: 10.3390/nu16213707 (PMC11547887; doi:10.3390/nu16213707)
Supplement: Supplementary file 1 [file nutrients-16-03707-s001.zip › Table S3.pdf]

**Table S3.** Characteristics of participants in the pilot study of a self-assessment instrument for the lifestyle of breast cancer survivors (PrevCancer) according to tertiles*.

| Variable | Sample Usability and Acceptability test | | | | Sample Convergent Validity test | | | |
|---|---|---|---|---|---|---|---|---|
| | System Usability Scale Score | | | | PrevCancer Score | | | |
| | Total (N=65) | Tertile 1 (score 0 to 82.5, n= 23) | Tertile 2 (score 85 to 95, n=23) | Tertile 3 (score 97.5 to 100, n=19) | Total (N=55) | Tertile 1 (score 3 to 6.0, n=24) | Tertile 2 (score 6.25 to 6.75, n=14) | Tertile 3 (score 7 to 9.75, n=17) |
| **Age (years)**[a] | - | 33.7 (3.3) | 28.9 (2.5) | 31.6 (2.9) | - | 39.2 (3.1) | 29.3 (3.3) | 31.5 (2.9) |
| **Education**[b] | | | | | | | | |
| 1st degree complete | 1 (100) | 0 (0) | 0 (0) | 1 (100) | 1 (100) | 0 (0) | 1 (100) | 0 (0) |
| Incomplete 2nd degree | 4 (100) | 2 (50) | 2 (50) | 0 (0) | 2 (0) | 2 (100) | 0 (0) | 0 (0) |
| Complete 2nd degree | 3 (100) | 2 (67) | 0 (0) | 1 (33) | 1 (100) | 1 (100) | 0 (0) | 0 (0) |
| Incomplete graduation | 28 (100) | 9 (32) | 11 (39) | 8 (29) | 26 (100) | 10 (38) | 8 (31) | 8 (31) |
| Complete graduation | 29 (100) | 10 (34) | 10 (34) | 9 (32) | 25 | 11 (44) | 5 (20) | 9 (36) |
| **Race/ethnicity**[b] | | | | | | | | |
| White, non-Hispanic | 47 (100) | 16 (34) | 19 (40) | 12 (26) | 38 (100) | 16 (42) | 10 (26) | 12 (32) |
| Black, non-Hispanic | 4 (100) | 2 (50) | 1 (25) | 1 (25) | 4 (100) | 2 (50) | 1 (25) | 1 (25) |
| Other, non-Hispanic | 14 (100) | 5 (36) | 3 (21) | 6 (43) | 13 (100) | 6 (46) | 3 (23) | 4 (31) |
| **Marital status**[b] | | | | | | | | |
| Married/stable union | 17 (100) | 5 (29) | 5 (29) | 7 (42) | 13 (100) | 4 (31) | 4 (31) | 5 (38) |
| Not married/no stable union | 48 (100) | 18 (37) | 18 (37) | 12 (26) | 42 (100) | 20 (48) | 10 (24) | 12 (28) |
| **Average monthly family income**[b] | | | | | | | | |
| Up to <5 minimum wages | 22 (100) | 10 (46) | 6 (27) | 6 (27) | 18 (100) | 8 (44) | 3 (17) | 7 (39) |
| Up to ≥5 minimum wages | 43 (100) | 13 (30) | 17 (40) | 13 (30) | 37 (100) | 16 (43) | 11 (30) | 29 (27) |

*Tertiles of System Usability Scale Score for Sample Usability and Acceptability test and tertiles of PrevCancer Score for Sample Convergent Validity test.
[a]Results presented in mean and standard deviation. [b]Results presented in n and %.
