# Supplementary material for: Development, Content Validity and Usability of a Self-Assessment Instrument for the Lifestyle of Breast Cancer Survivors in Brazil
Source: Nutrients. 2024 Oct 30;16(21):3707. doi: 10.3390/nu16213707 (PMC11547887; doi:10.3390/nu16213707)
Supplement: Supplementary file 1 [file nutrients-16-03707-s001.zip › Table S4 .pdf]

Table S4. Usability analysis (pilot study) of a self-assessment instrument for the lifestyle of breast cancer survivors (PrevCancer).

Usability attribute	n (%)	Key observations of the participants
**Appearance of the tool**		"Appearance did not affect the answers"
1 - I hated it	-	
2 – I did not like it	-	
3 - Indifferent	4 (6)	
4 - I liked it	24 (38)	
5 - I loved it	35 (56)	
**Difficulty in filling out the instrument**		"In the items referring to alcoholic beverages, I missed an alternative regarding sporadic consumption, as there are few response options, and I don't drink every week."
No	57 (88)	"It should be better explained how to calculate the minutes of physical activity practiced".
Yes	8 (12)	"I had difficulty identifying my type and level of physical exercise."
		"I only had difficulty understanding questions involving calculation, as I don't consider myself good at mathematics."
		"It was very easy to fill out the questionnaire."
**Understanding of the questions**		"The calculation of the weekly duration of physical activity was confusing."
1 - I hated it	-	"Regarding breastfeeding, I believe there could be an option of `I don't have children` for better understanding."
2 – I did not like it	1 (2)	
3 - Indifferent	2 (3)	
4 - I liked it	22 (35)	
5 - I loved it	37 (60)	
**Technical problem of the instrument**		"The form page crashed while filling out."
No	60 (92)	
Yes	5 (8)	
**Feeling when filling out the instrument**		"I was afraid of what the result of the score would be."
Calm	61 (94)	
Agitated	4 (6)	
Other	-	
**General experience of using the instrument**		"The questionnaire is a bit extensive."
1 - I hated it	-	"I would like to know in advance how many questions the form has and how long it takes to answer."
2 – I did not like it	-	"I would like to be able to compare the results of different applications of the questionnaire."
3 - Indifferent	1 (2)	
4 - I liked it	28 (44)	
5 - I loved it	34 (54)	

	"I suggest including the vegetarian/vegan option in the question about meat consumption, as the questionnaire would be more inclusive."<br>"It needs to be disclosed to other people. This system is very good."
